# Squamous Cell and Adenoid Cystic Carcinoma Collision Tumor of the Soft Palate Treated with Surface Mold Brachytherapy

**DOI:** 10.7759/cureus.7297

**Published:** 2020-03-17

**Authors:** Leonid Reshko, Zafrulla Khan, Keith T Sowards, Adrienne Jordan, Craig Silverman

**Affiliations:** 1 Radiation Oncology, University of Louisville School of Medicine, Louisville, USA; 2 Maxillofacial/Oncologic Dentistry, University of Louisville School of Dentistry, Louisville, USA; 3 Pathology, University of Louisville School of Medicine, Louisville, USA; 4 Radiation Oncology, University of Louisville, Louisville, USA

**Keywords:** oropharyngeal carcinoma, squamous cell carcinoma, adenoid cystic carcinoma, collision tumor, dual primary, immunosuppression, brachytherapy, head and neck cancer, head and neck pathology, superficial brachytherapy

## Abstract

Simultaneous primary cancers are rare in the oropharynx. This report describes the first reported case of a collision tumor of squamous cell and adenoid cystic carcinoma in the soft palate. The patient was immunosuppressed with a history of liver transplantation, smoking and heavy alcohol drinking. He was treated with wide local excision followed by adjuvant radiotherapy with surface acrylic mold brachytherapy. This technique was used instead of external beam radiotherapy in order to minimize toxicity. The patient tolerated the treatment well and with the only acute grade two mucositis at the soft palate and minimal late toxicity. There is no evidence of disease recurrence and the patient continues to maintain excellent quality of speech and swallowing 14 months after treatment completion.

## Introduction

The most common head and neck malignancy is squamous cell carcinoma (SCC) [1]. Most common risk factors include smoking and human papilloma virus (HPV) infection. Adjuvant treatment of invasive SCC of the oropharynx depends on the pathologic risk factors such as advanced disease, perineural invasion, lymphovascular space invasion and/or close margins (<5 mm) [2]. Adenoid cystic carcinoma is a rare tumor occurring in only 1% of head and neck cancers but represents 10% of all salivary gland neoplasms [3]. While the oral cavity and oropharynx salivary gland malignancies have a low propensity for cervical lymph node metastasis and neck irradiation is typically not indicated, adenoid cystic carcinomas often benefit from adjuvant radiation therapy due to their propensity for local failure and perineural invasion [3,4]. An improvement in survival has been shown even in early-stage disease with postoperative radiotherapy [5].

Immunosuppressed organ transplant patients are at a higher risk of developing secondary cancers. The incidence of any cancer is increased by a factor of 2.2-4.9 after liver transplantation [6-8]. The increase in risk for developing head and neck cancer after liver transplant was found to be 3.8 in a recent meta-analysis including developing unusual malignant histologies [9]. However, having both a squamous cell carcinoma and an adenoid cystic carcinoma in the oropharynx has never been reported [10,11]. In the head and neck region, one case report of a collision tumor of the larynx has been documented [12]. In the cervix, there is a 27-case series describing patients with coexisting invasive SCC and adenoid cystic carcinoma of the cervix. In this context, the coexistence of these tumors was hypothesized to be due to HPV infection [13].

External beam radiotherapy is used most commonly in the treatment of oral and oropharyngeal cancer and adenoid cystic carcinoma of the salivary glands. Adenoid cystic carcinoma, in particular, has a propensity for perineural spread which often cannot be addressed by more superficial techniques such as brachytherapy that treats superficial tissues but does not penetrate deeper [4,14]. However, external beam radiotherapy is associated with significant acute and late toxicity including mucositis, dermatitis and xerostomia as a significant amount of normal tissues is irradiated. Occasionally, brachytherapy is used in cancer centers with expertise in this modality to minimize toxicity in select cases of oropharyngeal and salivary gland cancers. The radiation dose to the normal tissues can be lowered by this method in select cases [15-17]. When tumors are technically accessible and superficially-located, surface mold brachytherapy technique may be utilized in oral and oropharyngeal cancers [7,8,18,19].

## Case presentation

**Figure 1:**
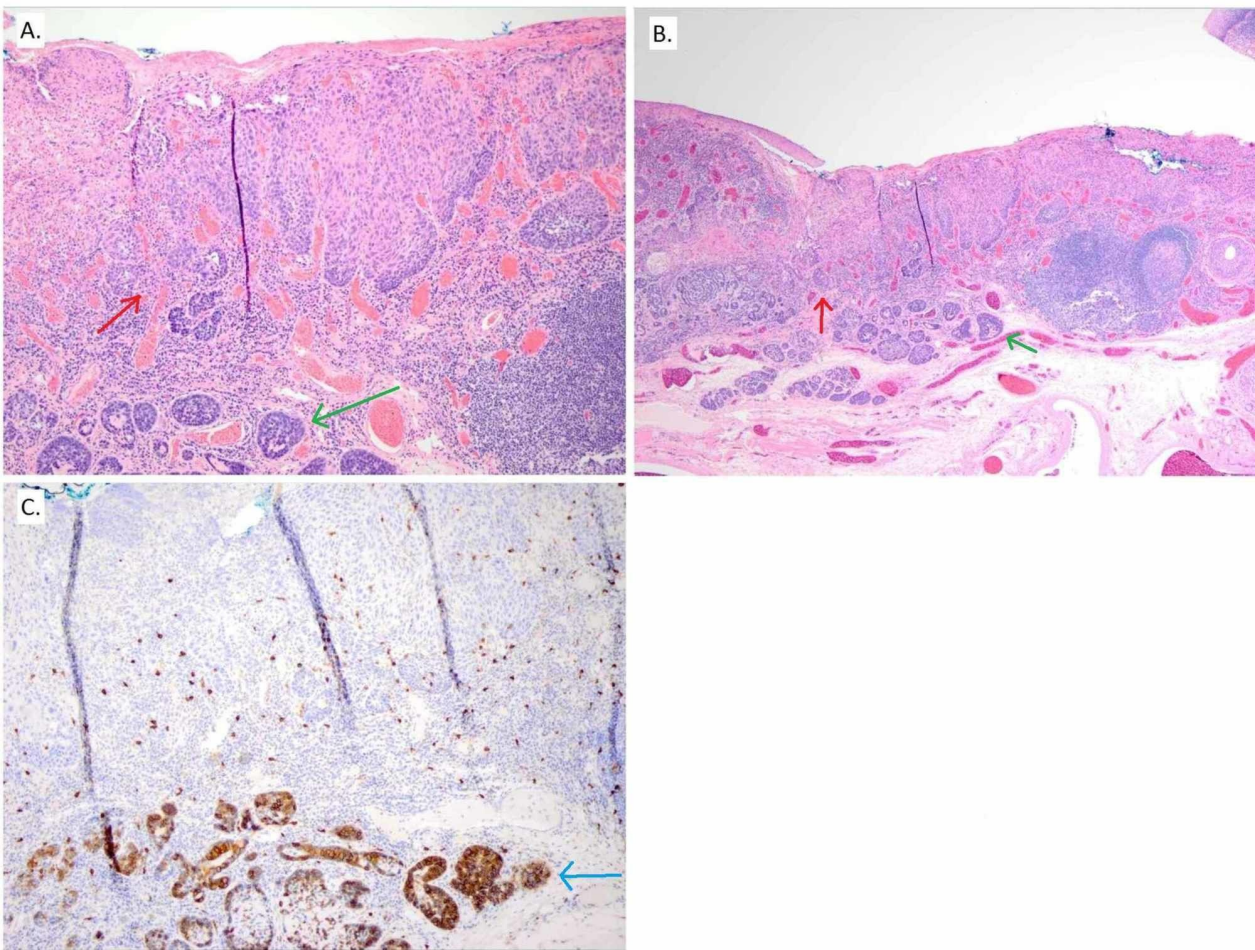
Pathological specimen. (A) Higher power Hematoxylin and Eosin (H&E) image. (B) Lower power H&E image. Both (A) and (B) show invasive squamous cell carcinoma with a red arrow and adenoid cystic carcinoma with a green arrow. (C) CD117 staining of subepithelial cells showing adenoid cystic carcinoma (blue arrow).

**Figure 2:**
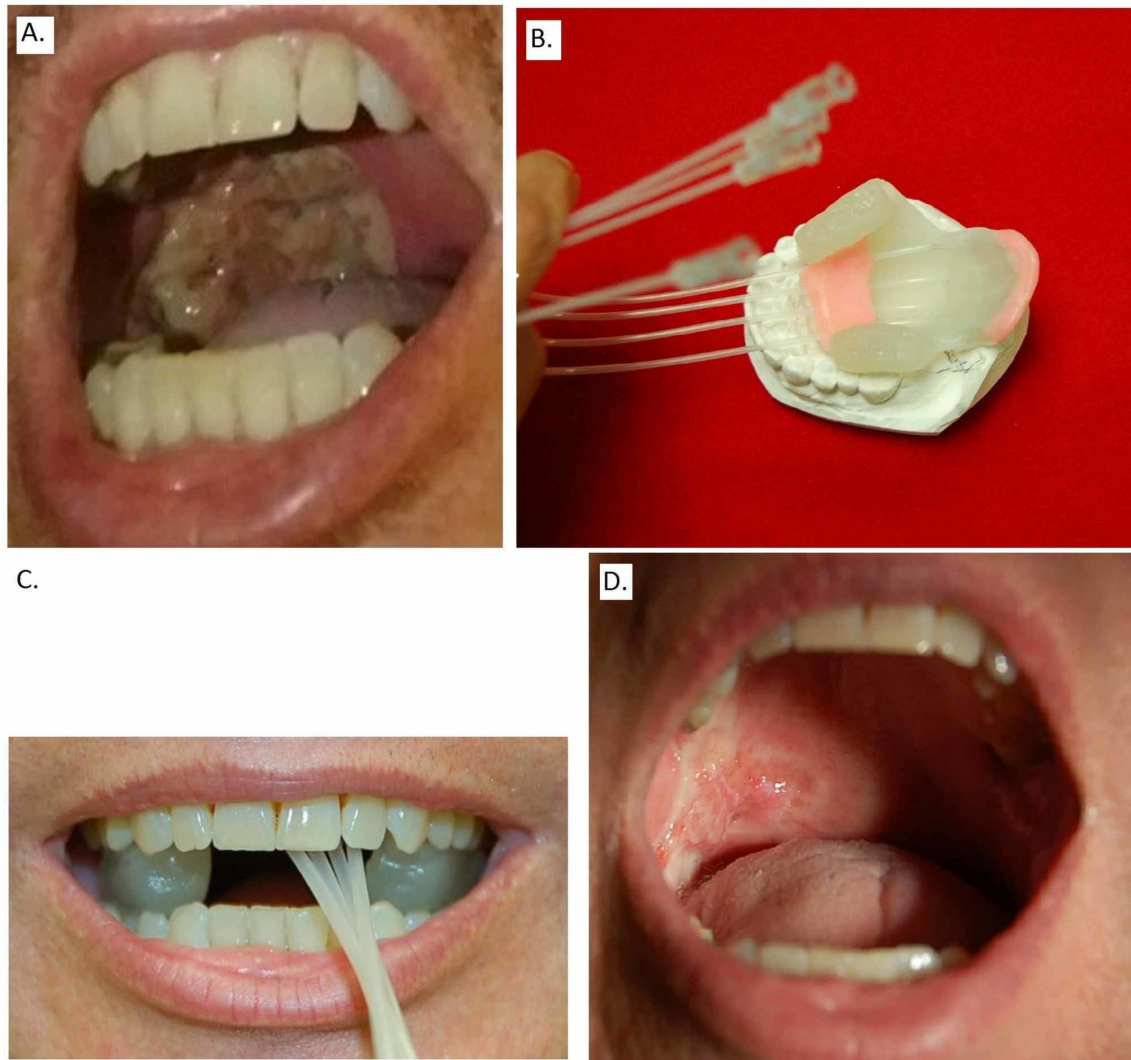
(A) Patient’s palate prior to treatment. (B) High-dose-rate (HDR) brachytherapy acrylic mold. (C) Device in place. (D) Appearance of the treated area one week after adjuvant brachytherapy.

The patient is a 48-year-old Caucasian male heavy smoker with a 30-pack-year history who also has alcoholic cirrhosis status post-liver transplantation in 2015. The patient has not had any significant health problems since his transplant. He has been on Cyclosporine since 2015. He presented in 2018 with a right soft palate mass originally noted by his dentist. There was erythroplakia with a lesion that was superficial in appearance and extended to the level just above the uvula but did not cross the midline and extended down the anterior tonsillar pillar involving the retromolar trigone but not the tonsil. Biopsy showed SCC in situ on the background of severe dysplasia. The resection was performed by a head and neck oncology specialist. It involved partial pharyngectomy with wide local excision of the right soft palate and bilateral tonsillectomies. The gross specimen and tissue slides were reviewed by a head and neck cancer pathology specialist. The pathology showed a collision tumor of invasive SCC and adenoid cystic carcinoma histologies. The tumor with full-thickness keratinocyte atypia with squamous cell carcinoma in situ (Figure 1A) with a small area of squamous cell carcinoma invasion abutted a small intermediate grade adenoid cystic carcinoma within the subepithelium (Figure 1B). Each invasive lesion measured 0.6 cm. The adenoid cystic carcinoma expressed SOX10, CD117 (Figure 1C), actin and p40. SCC was p16 negative. Margins were negative with the closest being 4 millimeters (mm) for SCC in situ and 3 mm for adenoid cystic carcinoma. There was no lymphovascular space invasion or perineural invasion. Both lesions were staged as pT1 N0 stage I per AJCC 8th edition. Figure 2A illustrates the appearance of the treated area at the time of the patient’s evaluation by a radiation oncologist.

**Figure 3:**
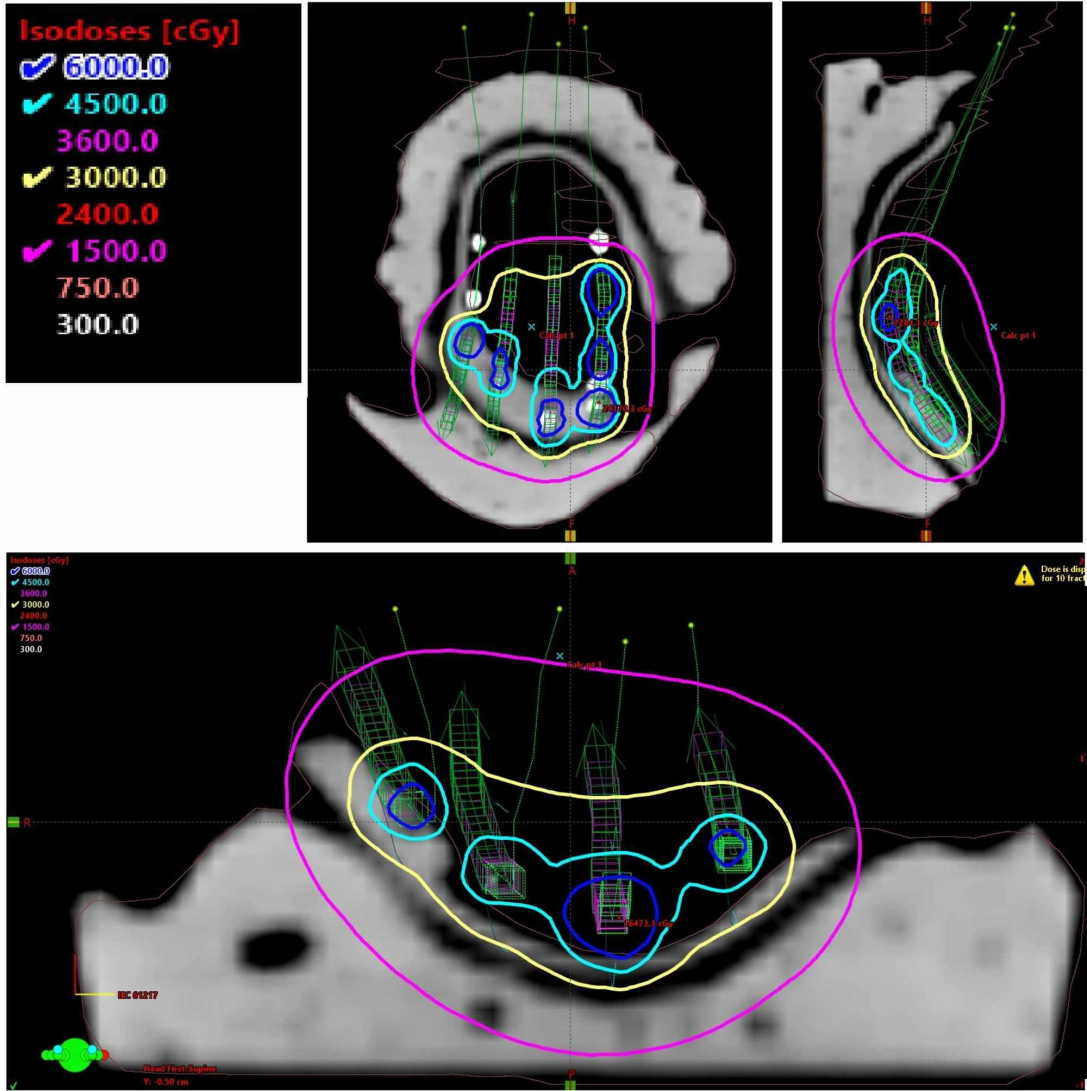
High-dose-rate (HDR) brachytherapy treatment plan with the isodose lines shown in axial, sagittal and coronal planes. Catheters and isodose lines are shown.

The case was extensively discussed at the multidisciplinary head & neck conference. There was a concern for local relapse due to close margins, the patient’s immune system compromise, and the fact that one of the tumors was adenoid cystic carcinoma. The patient was treated postoperatively with high-dose-rate (HDR) brachytherapy to address the possibility of microscopic residual disease and to minimize the toxicity of external beam radiation therapy. HDR was chosen over low-dose-rate brachytherapy as the latter technique was not shown to have improved outcomes, requires the patient to be hospitalized and exposes the staff to radiation [20]. An acrylic mold was fabricated by our dental oncologist with four afterloading catheters inserted 1 cm apart. Medical physicist and a radiation oncologist evaluated the design of the mold shown in Figure 2B. The patient was comfortable with the placement of the device as illustrated in Figure 2C. A local Lidocaine spray anesthetic was used as needed to ensure patient comfort and minimize the gag reflex. The setup was checked by the radiation oncologist and medical physicist for reproducibility and to ensure an absence of air gaps. An iridium-192 source was delivered via remote afterload technology through the catheters. The treatment time for each session was 80 seconds. The brachytherapy treatment plan is shown in Figure 3. A total of 30 Gy in 10 fractions calculated at 0.5 of a centimeter were delivered twice a day and were completed in one week. The soft palate V85 (volume in cubic centimeters (cc) receiving 85 Gy), V90, V100, and V150 were 0.72, 0.61, 0.42 and 0.09 cc, respectively.

The patient did remarkably well. Acute grade two mucositis was noted on the first follow-up the week after his treatment was completed. The patient had no xerostomia, odynophagia, difficulty speaking, problems with maintaining oral intake, and had minor residual pain that did not affect his quality of life. The appearance of the treated area on his first follow-up is shown in Figure 2D. Several months after treatment completion, he noted mild oropharyngeal discomfort, throat clearing and globus sensation. However, these resolved subsequently and he was asymptomatic on his last follow-up 14 months after treatment completion. The patient showed no evidence of disease recurrence.

## Discussion

Squamous and adenoid cystic carcinoma collision tumor of the oropharynx

Simultaneous primary malignancies are rare but have been well-described in the head and neck region [10]. Collision tumors are different from separate adjacent primary malignancies, metastatic and hybrid tumors in that the two malignant neoplasms arise at independent topographical sites and invade or collide into each other during growth, particularly in the border zones. This is distinct from biphasically differentiated tumors which are a repetitive mixture of two cellular patterns such as a sarcomatoid sarcoma [10]. Head and neck collision tumors that have been reported are hybrid salivary gland tumors such as acinic cell and adenoid cystic and dual primary neoplasms of the larynx, oral cavity, and hypopharynx [10-12]. The presence of both squamous cell carcinoma and adenoid cystic carcinoma in the head and neck region is extremely rare. Only one such case has been reported: a squamous cell carcinoma and adenoid cystic carcinoma collision tumor in the larynx [12]. To our knowledge, no malignant collision tumor of SCC and adenoid cystic carcinoma has been described to date in the oropharynx.

Diagnosis

The diagnosis was made based on a review of the H&E slides and immunostaining. The tumor p16 status was negative, so HPV virus infection did not contribute to the formation of this malignancy. The adenoid cystic carcinoma component expressed SOX10, CD117, actin and p40 as expected [3]. As SCC arises from the squamous epithelium and adenoid cystic carcinoma arises from the minor salivary glands, it appears that both of these malignancies occurred in the same tumor. In our patient, the development of both malignancies was likely contributed to by the patient’s immunosuppression [6]. In addition, the patient’s history of smoking and high alcohol intake may have contributed to the development of an invasive SCC component of his collision tumor [2].

Treatment

Finding an appropriate treatment of two T1a N0 oropharynx cancers in a collision tumor found incidentally is challenging. Given the small volume of disease, resection alone may be appropriate. However, given the adenoid cystic histology and the fact that surgical margins were close on both lesions, radiotherapy after surgery was favored to reduce the chance of local tumor recurrence [4,14]. External beam radiation therapy is typically used as adjuvant radiotherapy. However, this modality is known to cause significant toxicity in some patients including mucositis, xerostomia, odynophagia, fibrosis and osteoradionecrosis [2]. We utilized brachytherapy to minimize toxicity while achieving adequate coverage of the region at risk. Surface mold high dose rate brachytherapy was selected as this technique does not require an interstitial implant. This is a non-invasive procedure where an acrylic mold is manufactured with either hot sources or brachytherapy catheters placed in the device. Select cases of oropharyngeal and salivary gland cancers have been successfully treated this way. With adenoid cystic carcinoma, there is a concern for the tumor's propensity for nerve invasion. External beam radiotherapy may be used to address this. However, in our patient, the tumor was small in size, was located superficially and there was no perineural invasion, so we felt comfortable using brachytherapy [7,8,15-19]. The patient tolerated the procedure well with only grade two acute mucositis of the soft palate. To date, there is no evidence of disease recurrence, minimal late toxicity, and the patient continues to maintain excellent quality of speech and swallowing.

## Conclusions

In this report, we describe the first documented case of a collision tumor of squamous cell carcinoma and adenoid cystic carcinoma in the oropharynx. The two entities have different staining properties, and the slides show unequivocal collision and not hybrid tumors. The patient was treated successfully with surgical resection followed by the surface mold brachytherapy.
